
JOURNAL INFORMATION
==============================
NLM Title Abbreviation: Anim Front
Journal ID: Anim Front
NLM Journal ID: 101754918
Journal ID: anfron

Animal Frontiers: The Review Magazine of Animal Agriculture

ISSN: 2160-6056
EISSN: 2160-6064
Publisher: Oxford University Press

ARTICLE INFORMATION
==============================
PMCID: PMC12014273
DOI: 10.1093/af/vfaf002
Article ID: vfaf002
Article version: 1
Subjects: Society News, AcademicSubjects/SCI00960

News

Publication date: 2025 Apr
Electronic publication date: 2025 Apr 22
Volume: 15
Issue: 2
First page: 69
Last page: 75
Copyright: Copyright © 2024 American Society of Animal Science.
Copyright year: 2024
License: This is an Open Access article distributed under the terms of the Creative Commons Attribution License (https://creativecommons.org/licenses/by/4.0/), which permits unrestricted reuse, distribution, and reproduction in any medium, provided the original work is properly cited.
License URL: https://creativecommons.org/licenses/by/4.0/

Page count: 7

==============================

ANIMAL FRONTIERS

### Call for Papers for *Animal Frontiers* Themed Issues

Discover current open calls for papers open for members of the *Animal Frontiers* founding societies, American Society of Animal Science (ASAS), American Meat Science Association (AMSA), European Federation of Animal Science (EAAP), and the World Association for Animal Production (WAAP).

Submit your manuscript to these themed issues and disseminate your research to a global audience, featured on the Oxford Academic platform and with the founding societies.

For Review Only

### Themed Issue on One Health

Submission deadline: March 01, 2025

The intersection of human, animal, plant, and environmental health is of increasing importance. This issue will highlight the importance of and challenges to these interactions, and may include topics such as climate change, land use/cover changes, zoonotic diseases, and more.

### Themed Issue on Diversity, Equity, and Inclusion

Submission deadline: May 01, 2025

Diversity and equity in science and agriculture incorporates a wide range of perspectives, ideas, and solutions, leading to innovation. Ensuring that the diverse communities within agriculture are included allows those innovations to meet the needs of more communities globally. We invite papers addressing the ongoing efforts in addressing issues around diversity, equity, and inclusion in agriculture, including perspectives that share individual or group experiences in animal agriculture.

### Submit your paper

Each issue of *Animal Frontiers* consists of a series of invited, peer reviewed articles that present international perspectives on the status of a high-impact, timely, global issue in animal agriculture. The articles are in the form of discussion or position articles that will add to the scientific reviews and original articles published in the journals of the affiliated science societies.

Find more information about submissions, instructions to authors, and open access charges at https://academic.oup.com/af/pages/call-for-papers https://doi.org/10.1093/af/vfaf002

### Discover The Meat Locker

### Empowering Meat Processors with Resources and Expertise

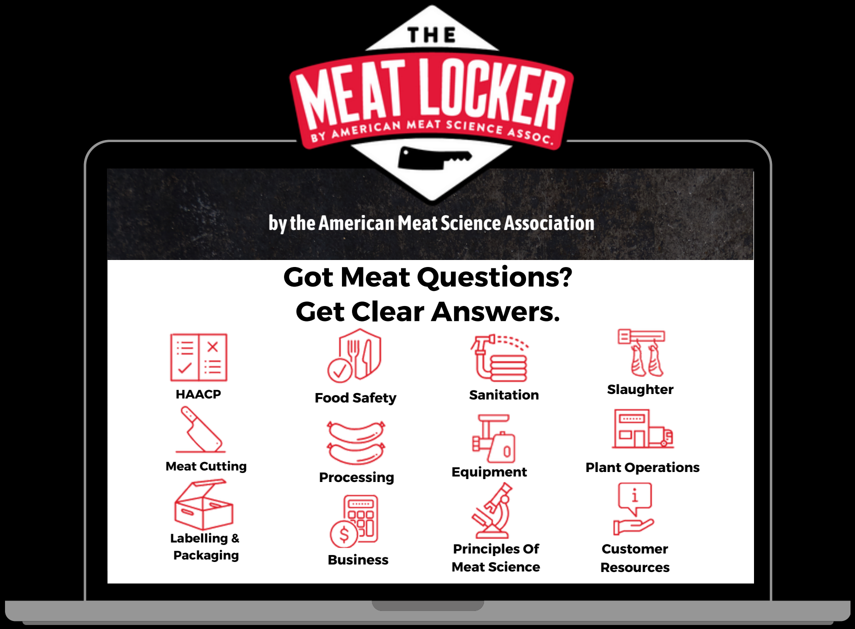

The American Meat Science Association (AMSA) is proud to offer The Meat Locker (TheMeatLocker.org), a dynamic new website designed to provide meat processors of all sizes with access to practical technical information, expert insights, and educational opportunities. Developed with simplicity and accessibility in mind, this platform addresses key topics such as processing, sanitation, HACCP, quality assurance, labeling, business operations, and more, making it an indispensable resource for the meat processing community.

**Visit "TheMeatLocker.org"**

Visit The Meat Locker today to explore its features and take advantage of these valuable resources. Together, we can support innovation, education, and success across the meat industry.

American Meat Science Association

*The Meat Locker was made possible through AMSA being a recipient of a USDA - Meat and Poultry Processor Technical Assistance (MPPTA) provider grant. For more information about AMSA, please visit https://meatscience.org*

https://mc.manuscriptcentral.c[illegible]

AMERICAN SOCIETY OF ANIMAL SCIENCE

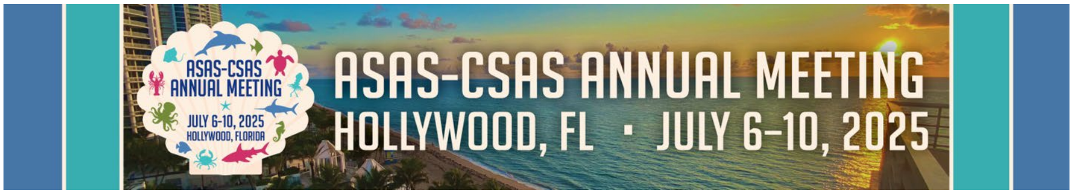

The 2025 Annual Meeting will take place at the Diplomat Beach Resort in Hollywood, Florida. When not in sessions, you are just steps away from soaking up the breezy South Florida lifestyle at the lagoon pool with ocean views of the alabaster beach.
Make sure to bookmark the meeting website, https://www.asas.org/meetings/annual-2025, to stay updated.
Abstract deadline: March 18, 2025

For Review Only

AMERICAN SOCIETY OF ANIMAL SCIENCE MIDWEST

The 2025 Midwest Section Meeting will be held in Omaha, NE, March 9-12, 2025.
Make sure to visit the Midwest Section Meeting website or scan the QR code below for more meeting information.

AMERICAN SOCIETY OF ANIMAL SCIENCE SOUTHERN | AMERICAN SOCIETY OF ANIMAL SCIENCE WESTERN

The ASAS Southern Section and ASAS Western Section will meet jointly in 2025 in Arlington, Texas, April 6-8, 2025. More information can be found on the meeting website, https://www.asas.org/meetings/2025-southern-western-joint-meeting
Abstract deadline is January 9, 2025.

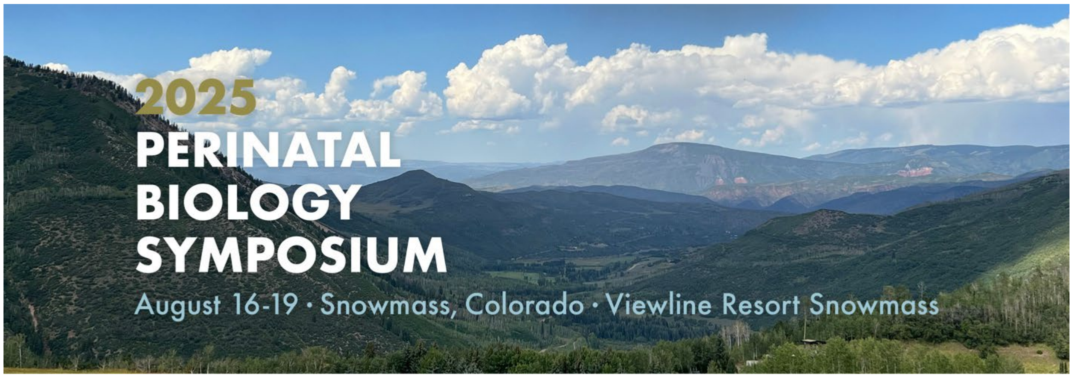

CHALLENGES AND SOLUTIONS FOR OPTIMIZING LONG-TERM HEALTH

Join us at the top of picturesque Snowmass Village, Colorado at the Viewline Resort Snowmass for the 2025 Perinatal Biology Symposium, August 16-19, 2025.

This Symposium will bring together clinicians, scientists and established senior and junior investigators from clinical medicine, basic research and applied livestock production, from around the world to report and discuss their findings in an atmosphere conducive to frank yet amicable exchange. Scientific exchange will occur across all levels via plenary sessions, poster sessions, trainee workshops and informal discussions, with time also dedicated to presentations by and recognition of trainees and early career investigators.
More information can be found on the meeting website, https://www.asas.org/meetings/perinatal-biology-symposium-2025

For Review Only

As a member of ASAS, we are sure you are aware of all the benefits that having a professional membership offer, including complimentary registration to monthly webinars and early access to the recordings, access to ASAS Journals, and discounted meeting registration to name a few. Remember to renew your ASAS membership or join ASAS to take advantage of these benefits.

More information on memberships can be found on the Membership website, https://www.asas.org/join-asas/individual-membership.

**EAAP**
**European Federation of Animal Science**

THE 76th EAAP ANNUAL MEETING WILL BE HELD IN INNSBRUK (AUSTRIA) FROM 25th TO 29th AUGUST 2025

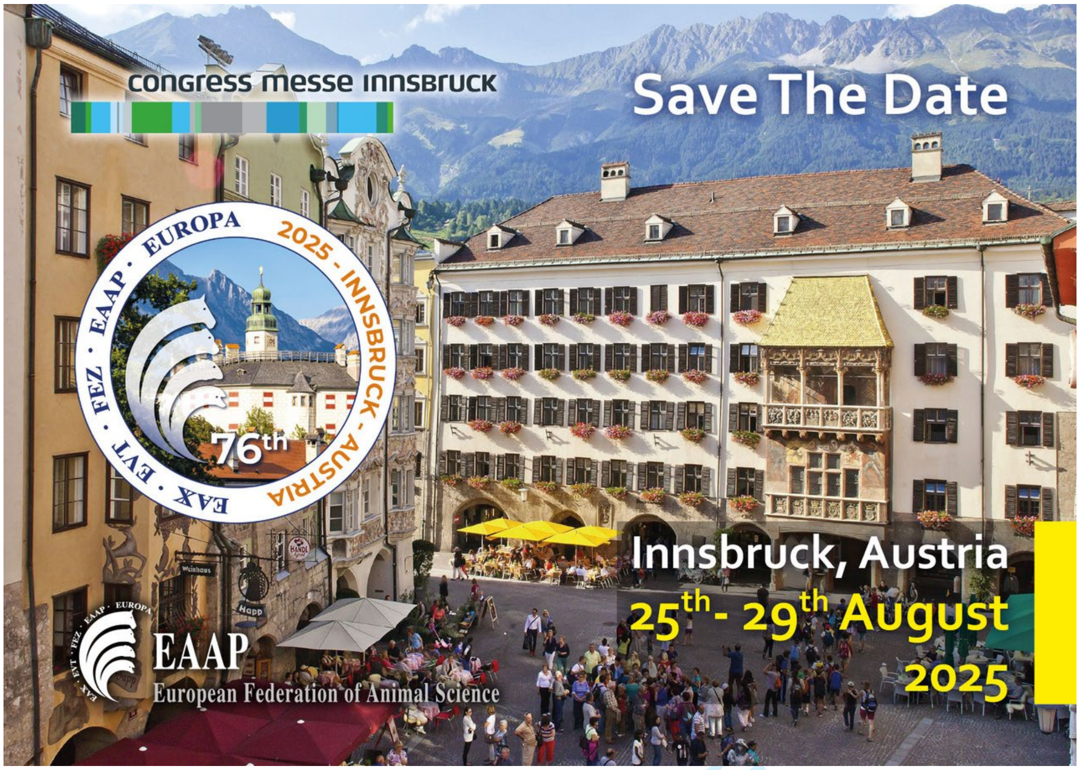

On behalf of the Austrian Organising Committee, we are very delighted to invite you to attend the 76th EAAP Annual Meeting 2024 to be held in Innsbruck (Austria), from 25th to 29th August 2025.
The sessions will be organized and jointly organized between the eleven EAAP Study Commissions to cover various areas of knowledge related to animal science, such as genetics, nutrition, physiology, animal health and welfare, precision livestock farming, horses, pigs, insects, livestock farming systems, cattle, sheep and goat. **Congress Website:** http:// www.eaap2025.org

**THE ABSTRACT SUBMISSION IS OPEN!**
Abstracts information https://eaap2025.org/abstracts-information/
All authors wishing to present papers at the EAAP Annual Meeting are required to submit the title and abstract of their presentations using the online application OASES.
The deadline for submission of the abstract will be March 1st 2025. Only abstracts submitted using the online application are considered for publication. Authors will be informed by mid-April 2025, whether their abstracts have been accepted for presentation during the meeting.
Authors, who submit abstracts, but fail to register and pay for the Conference by the 31st May 2025 will have their presentations removed from the program and their abstracts will not be inserted in the Abstract Book.

Please follow us on Facebook and Twitter
https://www.facebook.com/EAAP.ORG

INDIVIDUAL MEMBERSHIP OF EAAP

The EAAP membership is open to all scientists. It is a great opportunity to be update on the latest publications and other relevant information in the animal sector.
We have more than **6200 members** already!
Become a member of the most exciting international animal science network, joining us at
http://www.eaap.org/Content/Individual_Member_Information.html

THE EAAP NEWSLETTER FOR INDIVIDUAL MEMBERS IS NOW AVAILABLE ALSO IN CROATIAN, FRENCH, GERMAN, PORTUGUESE, ROMANIAN, SLOVENIAN, SPANISH, SLOVAK, ITALIAN AND POLISH!

For

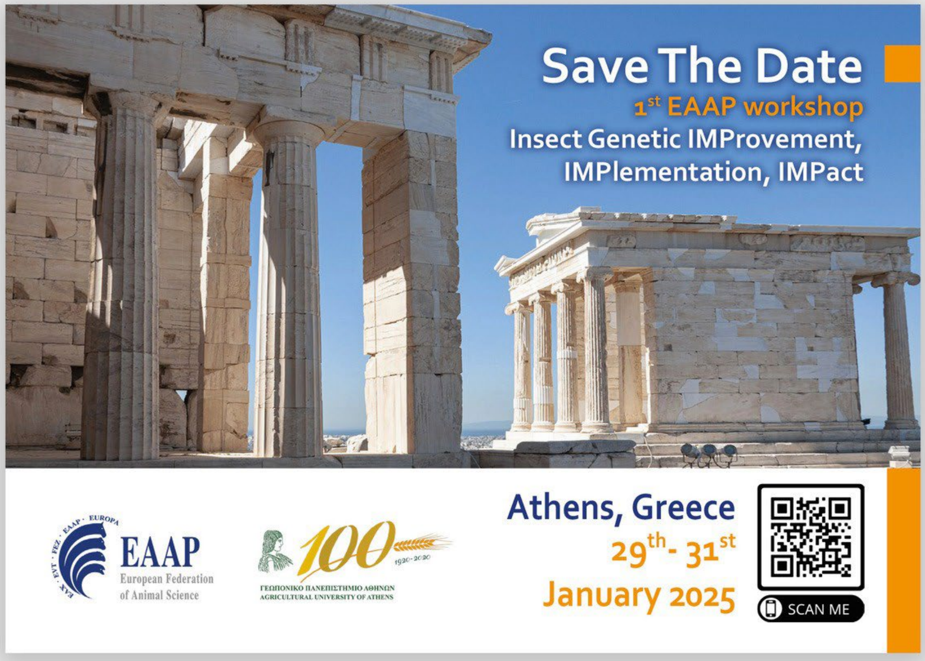

The World Federation of Animal Science Societies and Associations, **WAAP**, is an International Non-Governmental Organization with special consultative status with FAO since 1974.
The WAAP was established in 1965 after the success of the 1st World Conference on Animal Production held in Rome in 1963 organized by EAAP under the patronage of FAO.
WAAP aims to encourage closer collaboration between the animal production organizations and to bring together scientists, educators, technicians and administrators with the objective of reviewing development concerns on the five continents.

The Lyon city at dusk

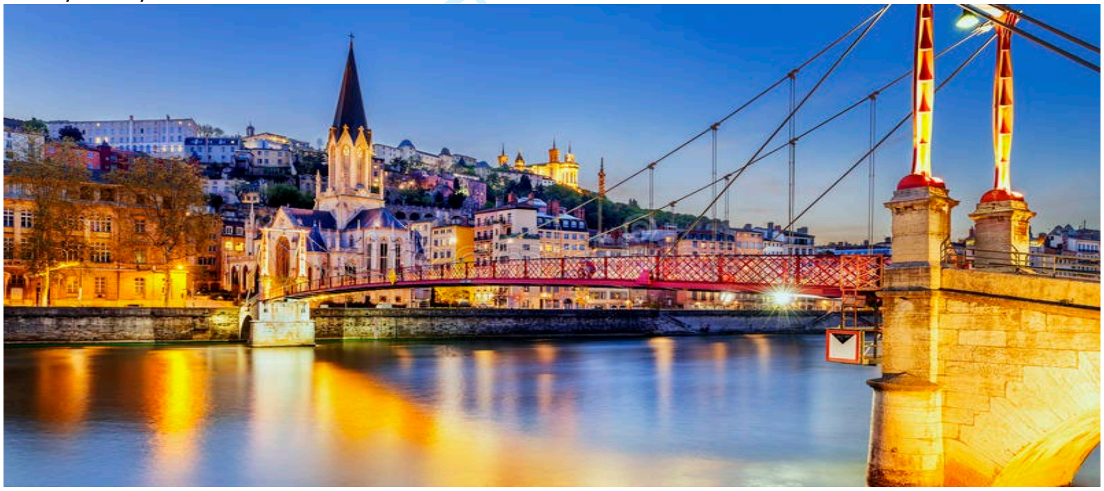

WAAP organizes every five years a World Conference on Animal Production. Co-organized by EAAP and WAAP, the 2023 conference on "Climate change, biodiversity and global sustainability of animal production", has been held in Lyon end August. It received 1900 abstracts among which 1200 were selected for oral presentations in 96 sessions. More than 2200 delegates came to the conference. Ten sessions, organized or co-organized by WAAP, were video recorded and will be soon available by streaming on the EAAP YouTube channel. Two of them were especially attractive on the "Control of GHG emissions in ruminant farming" and on "Biodiversity as a lever for sustainable animal production".
During the conference, WAAP delivered senior and junior awards and 3 student grants.
Philippe Chemineau ended his mandate after 5 years of presidency which was marked by the very positive entrance of WAAP in the Animal Frontiers consortium, but also negatively impacted by the COVID crisis.
Franck Dunshea was elected as the new President for 5 years. He will be in charge of organizing the
